# Progress in Medicine

**Published:** 1898-05-14

**Authors:** 


### PROGRESS IN MEDICINE.

### DISEASES OF THE HEART AND CIRCULATION.

(*Continued from page* 93).

**Infective Endocarditis.**—Kanthack and Tickell[13] have recently analysed the records of a large number of cases of infective endocarditis, more particularly with reference to its etiology. They find that micro-organisms may cause acute endocarditis without ulceration, and, conversely, ulceration may be found in their absence. Old cardiac disease was present in 64 per cent. of cases. Of all acute diseases pneumonia is most frequently complicated with endocarditis. When the pneumonia is croupous, the pneumococcus is the cause of the endocarditis, and seems to be attracted and arrested by a pre-existing cardiac lesion. Endocarditis is by no means common in typhoid fever. When it occurs, either streptococci or other pyococci, or typhoid bacilli have been found in the heart's blood. Similarly in those rare cases of infective endocarditis occurring in diphtheria or gonorrhœa, the Klebs-Löffler bacillus or Neisser's coccus may be present, or streptococci and other pyococci. Mixed infections are common in all these cases. Hence, when an endocarditis complicates an infective fever, it may be homologous or heterologous, or due to a mixed infection. Thayer has also shown that in some cases of endocarditis complicating gonorrhœa it is possible to obtain the gonococcus in pure culture from the blood stream.

Washburn[14] has recorded a case of ulcerative endocarditis successfully treated by antistreptococcic serum. The patient, who came under observation a few days after commencement of the illness, suffered during seven weeks from an ill-defined septicæmia without any local manifestation of disease. The pulmonary valve becoming affected, the serum was injected, with the result that the general condition improved, and the fever subsided, and the patient, except for the pulmonary regurgitation, perfectly recovered.

**Tuberculous Endocarditis.**—Etienne[15] has made a study of the occurrence of endocarditis in tuberculous affections. He finds that an acute form of inflammation of the cardiac valves is sometimes found associated with tubercle. Careful examination of these cases, however, throws doubt on the specific origin of many, for it is possible for se t:c conditions, such as are frequently found in the later stages of tuberculous disease, to produce endocarditis; and microscopic examination shows the absence of tubercle bacilli in the vegetations, and the presence of various forms of bacteria which are met with in other forms of endocarditis. In very rare cases, however, caseous foci may be discovered in the vegetations, and in these ordinary methods of staining reveal the presence of Koch's bacillus. Tuberculous endocarditis is then a most exceptional phenomenon.

**Angina Pectoris.**—Musser[16] points out that when dilatation of the heart supervenes in a patient the subject of attacks of angina pectoris, the subjective symptoms subside. At the same time the physical type of the individual changes from that of health to a pallid, pinched, frail condition. Angina may occur in a patient who has had dilatation of the heart, when this dilatation is removed by treatment. When true angina occurs in dilatation of the heart, it admits of a more favourable prognosis than when it occurs with other mural conditions, such as myocarditis or hypertrophy without dilatation. In dilatation of the heart, conversely, a better prognosis may be given if angina pectoris has previously occurred. These clinical facts suggest that the pain of angina is often due to increased intraventicular tension, and that the subsidence of pain, when dilatation comes on, occurs because of incompetency of the valves permitting of relief to the tension.

**Heart Failure of Arterio-sclerosis.**—Marshall[17] has recently discussed the treatment of the heart failure of arterio-sclerosis more particularly with reference to the combination of a vaso-dilator with digitalis. In the early stages of arterio-sclerosis the arteries are thickened and the heart is hypertrophied, with the result that the arterial blood pressure is increased, whilst in the terminal stage the hypertrophied heart dilates under the increasing pressure. In all the earlier stages probably no treatment is so useful as the iodides, together with an occasional mercurial purge, and vaso-dilators or digitalis are quite unnecessary. In the terminal stage of dilated heart the question of the vessel-contracting influence of digitalis becomes of importance. In some cases we find the pulse of low tension, although the arteries have thick walls, and in such cases digitalis alone, at least in the early stages, is by no means harmful. But in the majority of cases the arterial factor is an important one. It is important then to be able to combine digitalis with a vaso-dilator. Experimental observations show that the two groups of substances are mutually antagonistic; as far as the blood pressure of animals is concerned digitalis diminishes the effect of nitro-glycerine, and in part nitro-glycerine counteracts the effects of digitalis. The only indication for digitalis is a failing heart, and the only indication for vaso-dilators an incompressible pulse. When the two conditions are combined, when a comparatively high tension pulse exists with heart failure, the combination of digitalis and a vaso-dilator will be of service. In doing so it must be remembered that whilst digitalis acts very slowly, all vaso-dilators act rapidly. Of the latter erythrol tetra-nitrate acts most slowly and for the longest time, but even in the case of this drug the action begins in twenty or thirty minutes, and lasts seven or eight hours, whilst the most soluble preparation of digitalis will not produce an action on the pulse under four or six hours. Marshall suggests that erythrol tetra-nitrate should be given every six hours, and one of the French granules of digitalin every one, two, or three days, according to the progress of the disease, and the effects of previous treatment.

[13] Kanthack and Tickell, Edin. Med. Jour., 1897. [14] Washburn, Lancet, Sept. 18, 1897. [15] Etienne, Archiv. de Méd. Exper., Jan., 1898. [16] Muser, Amer. Jour., Sept., 1897. [17] Marshall, Brit. Med. Jour., Dec., 1897.

### DISEASE OF RESPIRATORY ORGANS.

**Phthisis Tuberculin.**—Numerous reports are to hand on the results of the new R. tuberculin. T. Dutton Steele[1] recently reviewed them as follows: The new product is not more harmful than the old if carefully given and uncontaminated, but many samples are not pure, and some are stronger than others. The increase in the doses proposed by Koch is too severe, and the point of injection may become the seat of an abscess. The effects on existing tuberculous lesions are not yet certain, but in lupus and some other cases the remedy seems of some value. Koch shows that immunity could be brought about in guinea-pigs, and some patients no longer react to the old tuberculin after a course of the R. product, but it is unknown whether they are proof against an invasion of the bacteria themselves. A most serious point is the presence of various micro-organisms, and, apparently, even living tubercle bacilli in some specimens. Huber[2] treated 15 cases of phthisis under Von Leyden's care, and reported that, of four really suitable ones, two improved markedly, one was unchanged, and one nearly cured but relapsed. On the other hand, in five advanced cases two were possibly injured by the treatment. The course of injections could only be carried out to the end in the four first cases. Bussenius[3] analysed 34 cases, 29 being phthisis with or without other lesions. Out of them all, nine were much improved, seven were somewhat better, eight were unchanged, and ten became worse, or died. The treatment may be recommended in lupus, lenticular ulceration of larynx, "scrofula," early non-febrile phthisis, and, if the patient wishes it, in non-febrile phthisis with cavities, but on no account in febrile phthisis or in mixed infections, or if the patient loses ground when it is tried for a while. If success is met with, moderate injections should be given for a long while at intervals. Kernig[4] is more unfavourable, and reports that in five out of nine cases of phthisis the patients' condition was worse after the course than before, and the others do not seem to have improved. Strict aseptic precautions were used, and no abscesses developed at the point of injection. Moreover, he points out that the mild, suitable cases improve greatly under other treatment. His patients suffering from surgical tuberculosis did not benefit any more than the phthisical ones.

In a discussion at the Charité Hospital, Berlin,[5] it appeared that the drift of opinion was all against the remedy, and this view was shared by other medical societies there. Indeed, in the year which has elapsed since its discovery it has almost entirely failed to sustain the hopes which it at first excited.

**The Antiphthisin** of Klebs is, on the other hand, strongly recommended by T. H. Williams,[6] who claims thirty absolute recoveries under its use in two years at Ashville Sanatorium. S. L. Anderson and Allen are also quoted as having excellent results. Trudeau[7] has grown the tubercle bacilli for six years on artificial media with a view to obtain an attenuated, harmless species. He has inoculated animals with them, and nearly all survive. Finally, he has inoculated the survivors with virulent cultures, and finds that if not complete immunity at least an extraordinary prolongation of life has been given by the attenuated injection. The experiments go a long way to prove the possibility of creating immunity in men, a possibility which the ordinary chronic course of human tuberculosis, unlike that of the self-limited duration of the exanthemata, has rendered doubtful.

A. Salter[8] has shown that the sweat of phthisical patients contains large quantities of tuberculin, and when injected into animals gave a typical reaction. Similarly, the sweat of pneumonia gave marked effects, while the sweat of healthy persons caused no pathological changes at all if in moderate quantities. Hence he regards the night sweats of phthisis as an effort to eliminate the poison, and one which ought not to be interfered with by drugs.

**Icthyol** has been given as a remedy in phthisis alternately with creosote by Le Tanneur,[9] who administered one or two grammes daily in capsules with great success. Improvement followed in forty-two cases out of fifty thus treated. The remedy was also of great value in bronchitis, dilated bronchi, and paroxysmal cough. Fränkel and Williams are reported also[10] as using it with great success, and as ranking it higher than creosote in value for consumptives. The drug is best given in capsules which do not dissolve in the stomach, though Branthonne[11] gives the icthyolate of ammonia with alcohol in water, and finds it has value in retarding or arresting the disease.

**Creosote.**—Chaumier,[12] while insisting on the value of pure air, finds creosote a "marvellous" remedy, if given in large doses. Indeed, the more the patient can take the greater chance is there of recovery; and doses of this size can only be given by the mouth in pills or cod-liver oil. Guaiacol seems to him to have no special advantages, while creosotal has all the advantages of creosote without causing dyspepsia. He gives a teaspoonful of it, either alone or in some palatable form, twice or three times daily, but, if necessary, it can be administered by hypodermic or rectal injection. In fever and diarrhœa he objects to any of these drugs, but Siefert[13] thinks their action beneficial in these states, only he advocates smaller doses, such as five drops of creosotal at a time, to be increased to twenty-five. He, however, agrees as to the superiority of creosotal, and mentions that in Germany it is no more expensive in these doses than emulsions of creosote. The latter is certainly cheaper in this country if simply beaten up in milk, as out-patients usually take it. Graham Crookshank[14] seems to have found at Brompton that guaiacol is the best, and prescribes it with rectified spirit and water. Heusser[15] of Davos has obtained good results from intra-muscular injections of cinnamic acid, commencing with $1\frac{1}{2}$ minims of a 5 per cent. emulsion, and increasing even up to 15 grains. Some burning pain is felt. and may last all day, but marked improvement takes place in many cases.

[1] Inter. Med. Mag., Dec. [2] B. Med. Jour., March 5, [3] B. Med. Jour.. April 2. [4] B. Med. Jour., March 19. [5] Med. News, March 19. [6] Bristol Med. Chir. J., Dec. [7] B. Med. Jour., Dec. 25. [8] Lancet, Jan. 15, [9] Gazette des Hospit., Jan. 5. [10] Bristol Med. Chir. J., Dec. [11] Post-graduate, Jan. [12] Lancet, Jan. 22. [13] Lancet, April 2. [14] Clin. Jour., Dec. 15. [15] Amer. M. S. Bulletin, Dec. 10.

